# Biodegradable magnesium alloy (WE43) in bone‐fixation plate and screw

**DOI:** 10.1002/jbm.b.34582

**Published:** 2020-02-12

**Authors:** Soo‐Hwan Byun, Ho‐Kyung Lim, Kwang‐Hee Cheon, Sung‐Mi Lee, Hyoun‐Ee Kim, Jong‐Ho Lee

**Affiliations:** ^1^ Department of Oral and Maxillofacial Surgery, Hallym University Medical Center Dongtan Sacred Heart Hospital Hwaseong Korea; ^2^ Department of Oral and Maxillofacial Surgery Seoul National University Dental Hospital Seoul Korea; ^3^ Department of Oral and Maxillofacial Surgery, Korea Medical University Medical Center Guro Hospital Seoul Korea; ^4^ Biomedical Implant Convergence Research Center Advanced Institutes of Convergence Technology Suwon Korea; ^5^ Department of Material Science and Engineering Seoul National University Seoul Korea

**Keywords:** absorbable plate and screw, biocompatibility, bone‐fixation plate and screw, magnesium, WE43

## Abstract

The purpose of the present study was to evaluate the mechanical strength and the absorption rate of WE43 material and to develop an absorbable metallic plate and screw for craniofacial application. The extruded WE43 plate and screw were evaluated using a LeFort I osteotomy canine model of 10 beagle dogs. Animals were divided into two groups: five dogs in the experimental group and five dogs in the control group. μCT was acquired at 4, 12, and 24 weeks. At 24 weeks after the operation, all animals were sacrificed, and histologic evaluation was performed. Swelling and gas formation were observed in three dogs in the experimental groups at 8 weeks. From 12 weeks, infraorbital fistula and inflammation were observed in three dogs in the experimental group, which gradually decreased and disappeared at 24 weeks. Other two dogs showed less gas formation at 12 weeks. The plates were completely absorbed, and gas formation was not observed at 24 weeks in these two dogs. New bone was well formed around the plates and screws in both groups. Histologic examination showed no specific differences between two groups. The mechanical strength of extruded WE43 was sufficient for mid‐facial application. Plates and screws made with appropriately treated WE43 have the potential to be useful clinically.

## 1 INTRODUCTION

Non‐absorbable titanium (Ti) plate and screw remaining permanently in the body may increase the risk of infection (Jacobs, Gilbert, & Urban, 1998; Puleo & Huh, 1995). Additionally, in children invagination of fixation device into cranial cavity or possibility of prevention of bone growth and damage to tooth, surgical removal of Ti fixation device is generally recommended after adequate bone healing time (Jacobs et al., 1998; Puleo & Huh, 1995).

As the polymer‐based resorbable bone fixation devices are absorbed into the surrounding fluid, the removal surgery is not needed. However, the strength of the polymer is still far lower than that of Ti. Polymer‐based devices are used only in limited areas that are not subjected to heavy forces (Dolanmaz, Uckan, Isik, & Saglam, 2004; Esen, Ataoglu, & Gemi, 2008). Furthermore, studies have reported long‐term foreign body reactions associated with the degradation of early polymeric devices, likely due to their acidic degradation products (Bergsma, Rozema, Bos, & de Bruijn, 1993; Bostman, Hirvensalo, Makinen, & Rokkanen, 1990; Weiler, Helling, Kirch, Zirbes, & Rehm, 1996). The foreign body reaction has been improved in the next generation polymer; however, the strength of that is still lower than that of Ti (da Silva et al., 2018).

Unlike Ti and polymer, Mg can provide a balance between strength and appropriate rate of degradation. Its elastic modulus and compressive strength are similar to those of human bone, so additional removal surgery is unnecessary (Liu et al., 2018). The Mg alloys that have been investigated as potential implant materials are rather complex in composition and contain potentially toxic elements (Witte et al., 2006). For alloys containing rare earth metal elements such as yttrium, their biological effects are still a matter of controversy (Gu, Zheng, Cheng, Zhong, & Xi, 2009; Witte et al., 2005). On principle, elements with potential toxicological problems should be avoided or used in minimal acceptable amounts for biodegradable materials manufacturing. Due to these reasons, Mg alloys should be evaluated for biocompatibility and stability (Lim et al., 2016). The purpose of this study is to develop an absorbable magnesium alloy plate with improved mechanical strength and evaluate the clinical feasibility for craniofacial bone fixation with in vitro and animal studies.

## 2 MATERIALS AND METHODS

### 2.1 Improving the strength of WE43 Mg alloy with extrusion technique

A WE43 Mg alloy rod was pre‐treated with an extrusion process to increase its strength (Daeryun Co., Shanghai, China) (extruded WE43) (Table 1). The alloy was homogenized at 525°C for 8 hr in an air furnace and pressed through the three‐roll planetary mill (PSW) at 400°C at a speed of 80 mm/s. The diameter of Mg alloy rod was decreased from 21 to 19.5 mm. After PSW treatment, the processed material was quenched in water and aged at 210°C for 16 hr. No lubricant was used during PSW procedure.

**Table 1:** Improved mechanical properties of extruded WE43

ASTM No.	Tensile strength (MPa)	Tensile yield strength (MPa)	Elongation (%)
WE43	260	160	6
Extruded WE43	303	195	6
Pure Mg	86	20	13
Ti alloy	715–1,050	693–950	10–28
Pure Ti	240–550	170–483	15–24

### 2.2 Biocompatibility and corrosion rate of WE43

The extruded WE43 Mg alloy rods were cut into disks with a diameter of 28 mm and a thickness of 5 mm. For evaluation of in vitro biocompatibility, the WE43 samples were grinded using SiC abrasive paper up to 2,000 grit. The other side of the WE43 samples was mounted with epoxy‐resin to prevent unnecessary corrosion. Every specimen was cleansed in ethanol, dried overnight, and sterilized in a clean bench with ultraviolet light for a day.

The biocompatibility of the WE43 alloy was evaluated using a pre‐osteoblast cell line (MC3T3‐E1). The pre‐incubated cells were seeded on disks at a density of 5.0 × 10^4^ cells/ml and cultured in α‐minimum essential medium (Welgene Co., Korea) by adding 10% fetal bovine serum and 1% penicillin streptomycin in a humidified incubator under an air atmosphere containing 5% CO_2_ at 37°C. After 1 day of culturing, the cell response was observed under Scanning Electron Microscope (SEM) (×1000, 20 kV, JSM‐5600, JEOL). Prior to the SEM observations, the samples were fixed with 2.5% glutaraldehyde for 10 min, dehydrated with graded ethanol (70, 90, 95, and 100% ethanol in sequence), immersed in hexamethyl‐disilazane for 10 min, and then air dried.

A long‐term static immersion test in simulated body fluid (SBF) was performed to observe the degradation behavior of the alloys over 60 days (Kannan & Raman, 2008). The tests were performed at a pH of 7.35 and 37.0°C in a thermostatic bath, and a 30 ml of SBF solution was used for each of the five disks. During the testing period of 60 days, the whole immersion system was regularly refilled with deionized water, and the conversion layer was not cleaned off. The remaining mass was measured once a week.

### 2.3 Mechanical properties of WE43

A three‐point flexural test was performed on a Ti plate (thickness of 0.5 mm), a pure Mg plate (thickness of 1.6 mm), casted WE43 (thickness of 1.2 mm), and extruded WE43 (thickness of 1.0 mm).

An Instron 8841 (Instron USA, Norwood, MA) with a 2‐kN load capacity was used with a constant bending punch speed of 1.0 mm/min and a support distance of 12.0 mm. ASTM F382 (specification and test method for metallic bone plates) was applied as the experimental protocol. For each sample, both ends of the plate were positioned on the same horizontal plane with two lower stabilizing points (Zaky et al., 2014). A tensile test was also performed on the Ti plate (thickness of 0.5 mm), the pure Mg plate (thickness of 1.0 mm), the casted WE43 (thickness of 1.0 mm), the extruded WE43 (thickness of 1.0 mm), and the extruded WE43 (thickness of 1.2 mm). Tensile strength was measured as the maximum stress that a plate could withstand while being stretched before breaking (Buijs, van der Houwen, Stegenga, Bos, & Verkerke, 2007).

### 2.4 Design of WE43 plate and screw for evaluation of strength and absorption rate

The extruded WE43 was used to fabricate the plates and screws. It was prepared in the form of rods. Screws were manufactured by machining with Computer Numerical Control (CNC) automatic lathes (Cincom L20, Citizen Machinery, Japan), and plates were manufactured by machining with CNC Machining Center (HSC 55, DMG Mori, Germany). The machined screws and plates were washed with sonicator for 5 min in 99.9% ethanol and dried with high pressure air. They were washed again in a clean room with the sonicator for 30 min in 99.9% ethanol and dried for an hour in an oven at 40°C. Finally, the screw and plates were individually packed into bottles and pouches, and sterilized with gamma irradiation (25 kGy, SAL 10^−6^).

**Figure 1:**
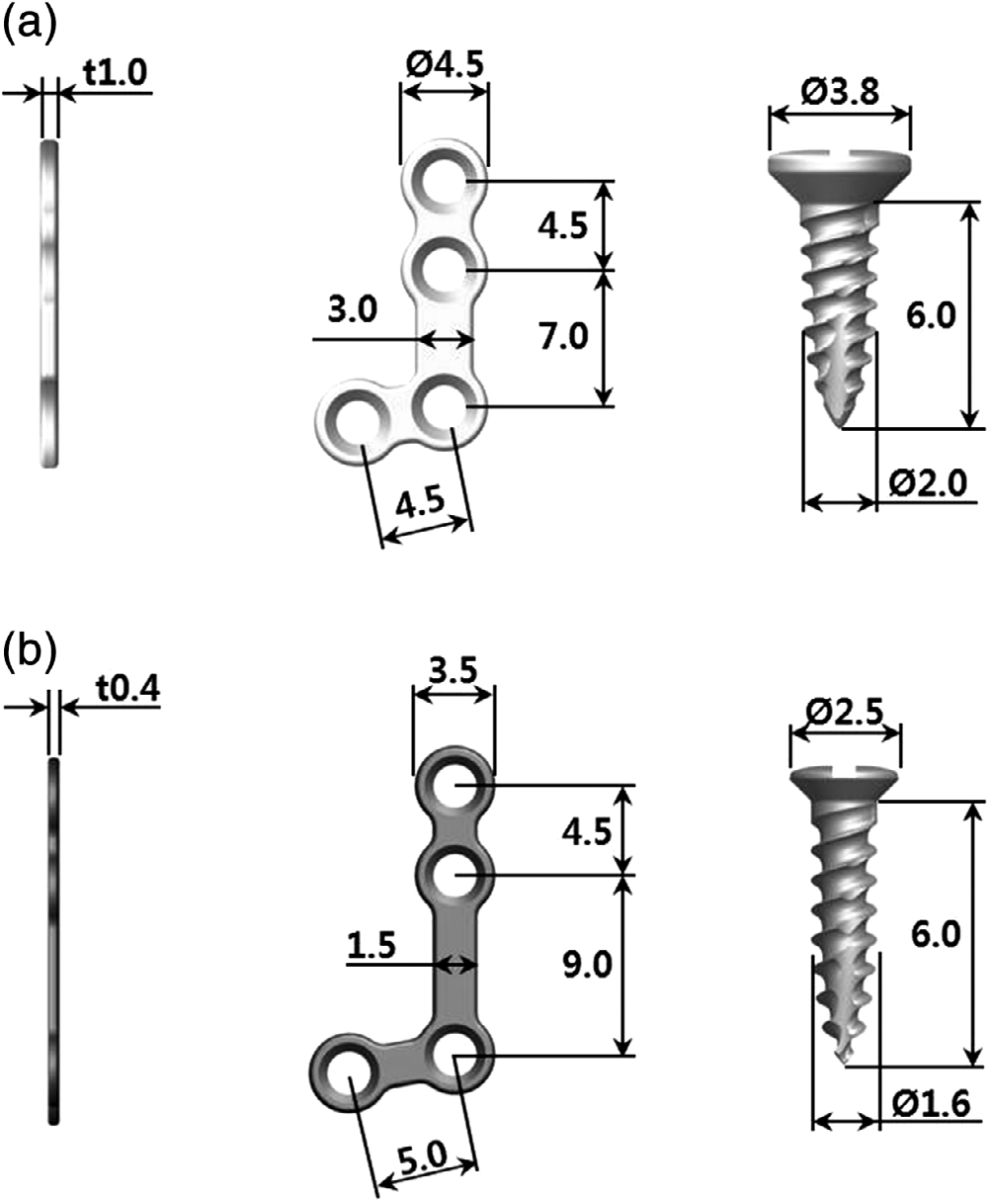
Design and dimensions of the plates and screws (WE43 and Ti). (a) WE43 magnesium alloy plates and screws; (b) Ti plates and screws

Figure 1 illustrates plates were made to be slightly thicker and larger than the Ti plates. The plates were designed to be L‐shaped with four holes. Using a milling machine, the plates were prepared with a 4.5 mm inter‐hole distance, 1.0 mm thickness, and 4.5 mm outer hole size. Screws with dimensions of 6.0 mm in length and 2.0 mm in diameter were prepared with a milling machine.

For the control group, Ti plates and screws were obtained from a DENTIUM CMF Plate System kit (Genoss Co., Suwon, Korea). Plates were prepared to be L‐shaped with four holes. Using a milling machine, Ti plates were prepared with a 4.5 mm hole distance, 0.4 mm thickness, 3.5 mm outer hole, and 1.7 mm inner hole. Ti screws with dimensions of 6.0 mm in length, 1.6 mm in diameter, and 2.5 mm in head diameter were prepared with a milling machine.

### 2.5 Preclinical evaluation of WE43 plate and screw using a LeFort I osteotomy canine model

This study was approved by the Seoul National University Institutional Animal Care and Use Committee (SNU‐160127‐6). Ten 20‐month‐old beagle dogs (10–15 kg body weight) were allowed a 4‐week adaptation period and then separated into two groups. WE43 plates and screws were used in the experimental group (five dogs), and the Ti plates and screws were used in the control group (five dogs). General anesthesia was administered intravenously. Block and infiltration anesthesia was performed intraorally with 2% lidocaine (Viscasillas, Seymour, & Brodbelt, 2013).

**Figure 2:**
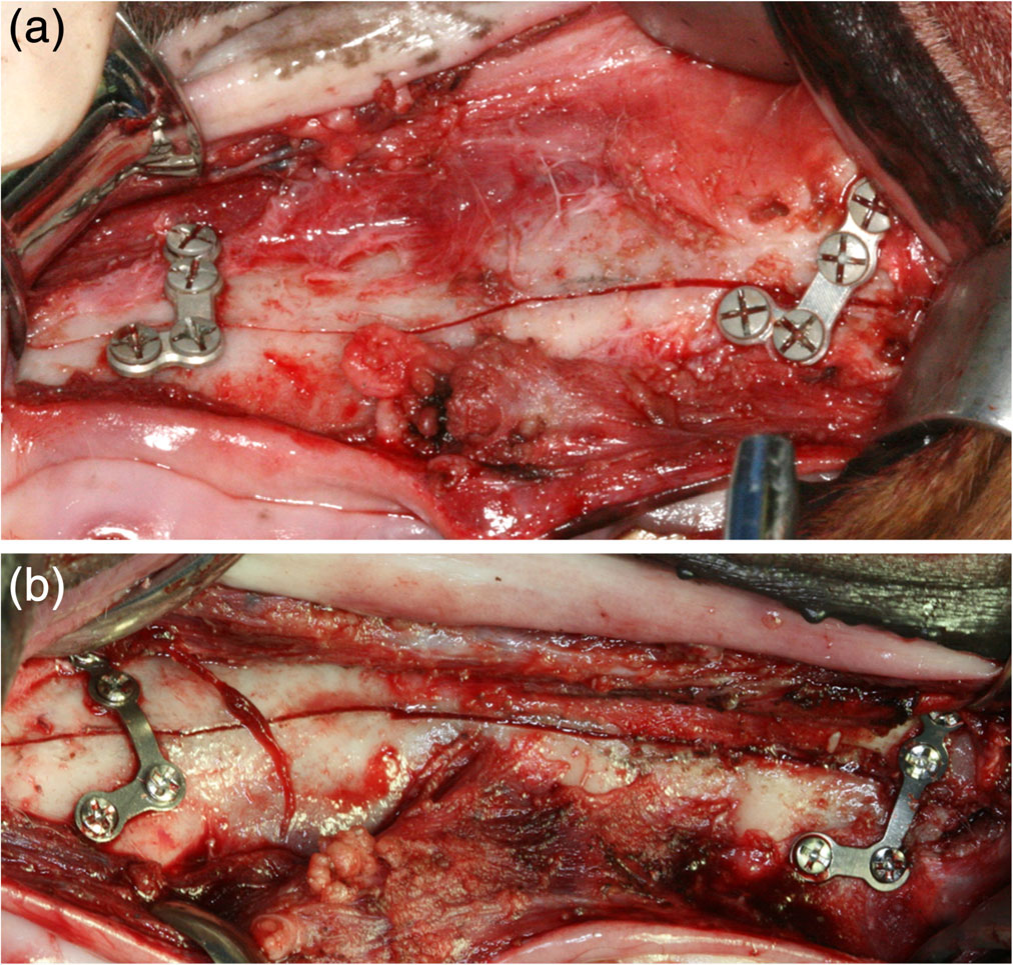
Intraoperative photograph of the fixation of plates and screws. Four plates and 16 screws were fixed. (a) WE43 magnesium alloy plates and screws; (b) Ti plates and screws

A sulcular incision was performed from the upper incisor region to the upper second molar region using a #15 blade. Periosteal elevation was performed posteriorly with minimal damage from the upper second molar to the maxillary tuberosity. The infraorbital nerve exposed during the periosteal elevation was further anesthetized using 2% lidocaine. The osteotomy line was planned from the top of the piriform aperture to the second molar apex. Four L‐shaped plates were fitted at the anterior buttresses on the canine site and the posterior buttress on the upper site of the third premolar (Zhao et al., 2009). After the plates were fitted, 16 holes were made on the bone using a 1.5‐mm drill (Dentium, Suwon, Korea) at a speed of 1,500 rpm with sufficient saline irrigation. Plates were fixed using 16 screws. Then, the four plates and 16 screws were removed. The LeFort I osteotomy was performed carefully, including the maxillary tuberosity area, using a reciprocating saw to ensure that the periosteum was not damaged. The separated maxillary bone was repositioned after hemostasis. Figure 2 showed four plates and 16 screws were fixed to the preformed holes. Copious irrigation and interrupted suture were performed using 4‐0 nylon sutures (Dafilon, Braun, Germany). Pulverized feed was given in the same amounts as before surgery for 4 weeks after the operation. Analgesics and antibiotics were mixed with the pulverized feed and were given for up to 7 days after surgery. After 4 weeks, the general diet was provided.

#### 2.5.1 *Clinical evaluation*

The presence of oral wound dehiscence, plate exposure, gas formation, inflammation, pus formation, occlusion, food intake, and fistula formation was evaluated weekly until 24 weeks. The mobility and stability of maxilla were evaluated by an examiner by holding the cranial bone with the left hand and the anterior portion of the maxilla with the right hand and shaking it back and forth. Mobility was classified into three groups: 2 = great mobility, 1 = little mobility, and 0 = no apparent mobility.

#### 2.5.2 *Evaluation of absorption rate using Micro Computed Tomography (μCT)*

μCT (SkyScan1173; SkyScan, Kontich, Belgium) was performed at 4, 12, and 24 weeks under general anesthesia. The absorption of the plates and screws and the change in the surrounding bone were evaluated.

In addition, the volume of the remaining screw fixed in the bone was calculated by combining 7.1 μm cross‐sectional cuts of μCT images of the remaining amount of the screw and the amount of cortical and marrow bone including 1 mm around the circumference of the screw and were constructed three‐dimensionally using CT analysis software.

#### 2.5.3 *Histologic evaluation*

At 24 weeks after the operation, all animals were sacrificed. After sacrifice, the maxilla was fixed in 10% neutral buffered formalin solution, decalcified, and stained with hematoxylin & eosin and Masson's trichrome (MT). The absorption of plates and screws, changes in the surrounding bone, inflammatory response, and bone formation were evaluated. The anterior and posterior positions of the plate were confirmed with the μCT image and were sectioned accordingly.

## 3 RESULTS

### 3.1 Biocompatibility and corrosion rate of WE43

There was no difference in cell attachment between pure Mg and WE43. The amount of mass loss with the WE43 samples during the static immersion test was observed as approximately 60% (60.07 ± 15.40) of the initial mass remained at 60 days.

### 3.2 Mechanical properties of WE43

In a three‐point flexural test, the results were different according to the type of material in the plate and its processing method. The flexural strength of WE43 was higher than that of pure Mg, and that of the extruded WE43 was higher than that of casted WE43. The outcome of the tensile test was similar to that of the three‐point flexural test. The tensile strength of the pure Mg plate (thickness of 1.0 mm) was lower than that of the Ti plate (thickness of 0.5 mm), but that of the casted WE43 plate (thickness of 1.0 mm) was higher. The extruded WE43 plate showed higher strength than the casted WE43 plate with the same thickness.

Based on these results, a 0.4‐mm Ti plate was used for the control group, and a 1.2‐mm Mg alloy plate was used for the experimental group. The strength of the Mg alloy was increased through an extrusion process, and the thickness was increased so that the strength was higher than that of the Ti plate in order to prevent fracture and tear.

### 3.3 Preclinical evaluation of WE43 plate and screw using a LeFort I osteotomy canine model

#### 3.3.1 *Clinical evaluation*

All dogs showed stable outcomes without any specific problems. After the operation, the stability of the maxilla was measured at “0,” the same as before the operation in all dogs. In the control group, there were no particular problems during the operation. On the 10th postoperative day, dehiscence was observed intraorally in the second dog of the experimental group and the fifth dog of the control group. Additional sutures were performed on the area. No dehiscence was observed intraorally until 24 weeks except for the dehiscence observed in the above mentioned dogs.

Swelling and gas formation were observed in three dogs of the experimental groups starting at 8 weeks. Infraorbital fistula and inflammatory symptoms were observed in three dogs of the experimental groups starting at 12 weeks, which gradually decreased and disappeared at 24 weeks. Occlusion remained stable until 24 weeks.

#### 3.3.2 *Evaluation of absorption rate using μCT*

**Figure 3:**
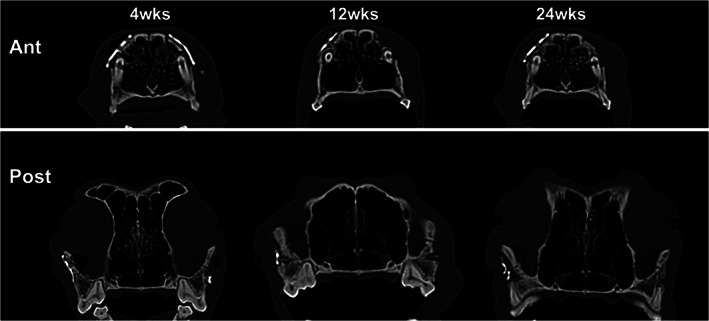
Radiological evaluation of the experimental group (WE43). Two dogs showed less gas formation at 12 weeks compared to other dogs. The plates were completely absorbed at 24 weeks in these two dogs. The LeFort I osteotomy line in the experimental group disappeared by 24 weeks

No specific findings were observed at 4 weeks in the experimental group. Gas formation was observed in three dogs of the experimental group at 12 weeks, but not at 24 weeks. All of the plates in the experimental group were well maintained until 12 weeks and mostly absorbed at 24 weeks. The plates separated from the bone and showed rapid absorption, but the screws fixed inside the bone showed slow absorption. Two dogs showed less gas formation at 12 weeks compared to the other three dogs. The plates were completely absorbed at 24 weeks in these two dogs. Figure 3 showed the LeFort I osteotomy line in the experimental group disappeared at 24 weeks. In the control group, plates and screws were maintained as at the initial position without any problem. The osteotomy line was clearly visible at 4 weeks and had slightly disappeared at 12 weeks. At 24 weeks, the osteotomy line was not observed, and complete bone healing was observed.

In the evaluation of the absorption rate using μCT images, all plates deviated from their original position as they were absorbed. It was difficult to overlay the plates. Thus, only 10 screws that remained in the original position were evaluated. As a result, 19.37% of the initial volume remained after 24 weeks. The new bone was measured to be approximately 10.97 mm^3^.

#### 3.3.3 *Histologic evaluation*

**Figure 4:**
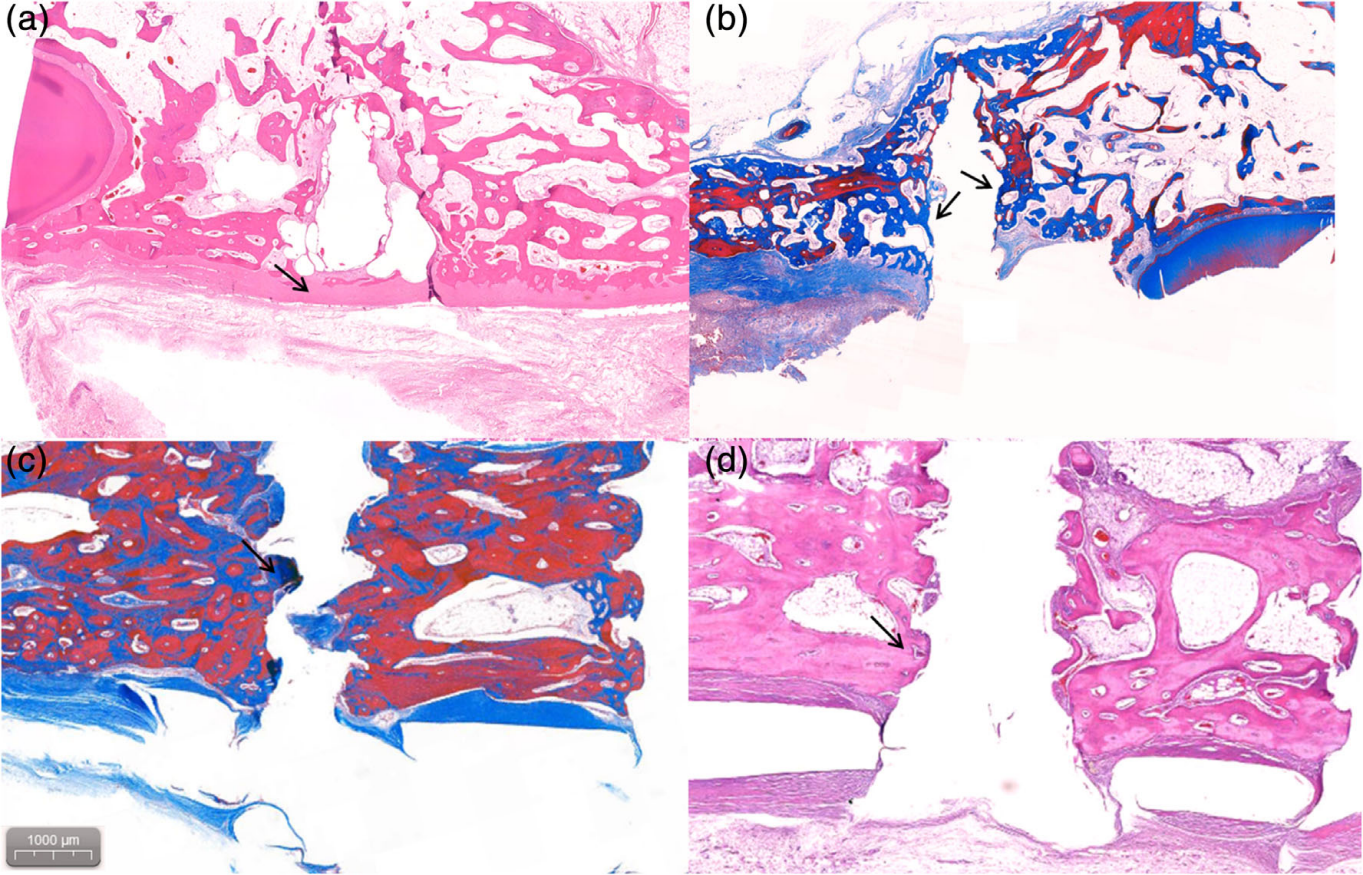
Histologic findings after 24 weeks (scale bar = 1,000 μm). New bone (black arrow) was formed near the screw head and covered the screw body in both groups. Histologic examination showed no specific differences between the experimental group and the control group. (a, b) Experimental group (WE43 plate); (c, d) control group (Ti plate)

Histologically, there was no inflammation in any of the groups. Soft tissue formation between the screw and bone was not found. An osteoblastic lining and woven bone were observed around the screw threads. New bone was well formed near the screw head and covered the screw body in the experimental group. New bone was well formed around the plates and screws, as seen in MT staining images in both groups. Figure 4 showed no specific differences between the experimental group and control group.

## 4 DISCUSSION

The extrusion process and changes in the design definitely increased the strength of the WE43 plates and screws (Liu et al., 2014; Zhang, Yuan, Niu, Fu, & Ding, 2012). Although additional tests should be performed to evaluate the biocompatibility and mechanical strength, these results demonstrate the possibility of WE43 for clinical application (Gu et al., 2010). The mechanical properties of Mg are similar to human bone; thus, the risk of stress shielding is mitigated (Byun et al., 2017). Furthermore, Mg has been shown to improve the osteogenic differentiation of cells and indirect bone formation (Hussain, Takahashi, Sonobe, Tabata, & Bessho, 2014; Webster, Ergun, Doremus, & Bizios, 2002; Yamasaki et al., 2003).

At the initial stage of corrosion, hydrogen gas was generated from the surface of Mg samples. It makes adherence of surrounding cells difficult on the surface of Mg sample, continuously (Kim et al., 2014). Generally, after adhering to the surface, cells can recognize surface morphology and chemical composition of materials through filopodia. Therefore, the formation of filopodia is enhanced if the adherence of cells is relatively simple. Through multiple filopodia, the interaction of cell‐material becomes active, and proliferation or differentiation of the cell occurs actively. However, the generated hydrogen gas makes it difficult for the cells to adhere to the surface. This reduced metabolism and consequently slowed down tissue regeneration (Mattila & Lappalainen, 2008; Meyer, Buchter, Wiesmann, Joos, & Jones, 2005). Furthermore, the accumulated hydrogen gas may attenuate bone formation. A large amount of hydrogen gas generated from the corrosion process was accumulated between the newly formed bone tissue and the surrounding soft tissue, affecting the acidity of the local environment and osteoblast activity. In addition, the space where the new bone tissue is to be regenerated was filled with hydrogen gas, which affects the quality of the regenerated bone tissue (Zhang et al., 2018).

In this present study, swelling was observed starting at 8 weeks, and hydrogen gas was likely generated. A large amount of hydrogen gas means that the absorption of Mg was too rapid. To overcome this, it is necessary to find a way to slow the absorption. Many methods have been studied to reduce the rapid corrosion of Mg such as alloying and surface modification (Li, Gu, Lou, & Zheng, 2008; Lorenz et al., 2009; Pietak, Mahoney, Dias, & Staiger, 2008; Remennik, Bartsch, Willbold, Witte, & Shechtman, 2011; Witte et al., 2005, 2006; Xin, Hu, & Chu, 2011; Xu, Yu, Zhang, Pan, & Yang, 2007). Surface modification with protective coatings can be applied to extend the absorption period, as mentioned above (Alvarez‐Lopez et al., 2010; Geng, Tan, Jin, Yang, & Yang, 2009; Gray‐Munro, Seguin, & Strong, 2009). Controlling the absorption rate means that the rate of hydrogen gas formation can be controlled as well (Byun et al., 2017). If the rate of hydrogen gas formation is slowed, the generated hydrogen gas can be absorbed into the body fluid, and swelling and fistula formation can be prevented. Control of the absorption rate using carefully engineered coatings has been described in other studies (Geng et al., 2009; Gray‐Munro et al., 2009; Xu, Zhang, & Yang, 2009).

According to a recently published paper by Lim et al. (2016), there was not a difference in the absorption rate of coated WE43 at 6 and 12 weeks. In reference to this study, the present study used non‐coated WE43 because the absorption can be too slow with the coating. However, there were some differences between the present study and the study of Lim et al. (2016). First, Lim et al. (2016) used screws only. If only screws are used (no plate), the total Mg volume is relatively small compared to the use of both screws and a plate. Therefore, hydrogen gas formation was lower than the present study. Second, the screw head was the first to be absorbed, and the plate was separated from the screw and bone in this study. As the separated plate deviated from its original position, there was increased contact with the body fluids, and absorption took place faster than with the screws that were fixed in the bone. Because of this, a larger amount of hydrogen gas was generated during the early stage. Third, screw placement was performed without osteotomy in the study of Lim et al. (2016), and LeFort I osteotomy was performed in the present study. When the osteotomy was performed, there was bleeding; as body fluid increased, absorption of Mg was accelerated. These could be the reasons for the differences between the results of Lim et al. (2016) and ours.

There are many ways to coat plates and screws, and HA coating has shown promise (Byun et al., 2017; Drynda, Seibt, Hassel, Bach, & Peuster, 2013; Hanzi, Gunde, Schinhammer, & Uggowitzer, 2009; Imwinkelried, Beck, Iizuka, & Schaller, 2013; Kim, Kim, Salih, & Knowles, 2005; Tan et al., 2014; Wong et al., 2010). HA coating increases the biocompatibility of Mg and contributes to bone formation by increasing osteoblast attachment (Faeda, Tavares, Sartori, Guastaldi, & Marcantonio, 2009; Kim et al., 2014; Ramires et al., 2003; Zreiqat et al., 2002). The first site of absorption was the screw head, and the site that remained until late was the screw body (Kim et al., 2014). This suggests that the body fluid of bone marrow does not actively absorb Mg, but the area directly underneath the mucosa seems to absorb rapidly due to external movement and dynamic flow of body fluid.

The size of the plates and screws should be adjusted based on our results. The Ti plates and screws used in the control group had sufficient strength to be used in not dogs but humans. In relation to the reduced size of the Ti plates and screws for dogs, the size of the Mg plates and screws for dogs can be reduced accordingly.

## 5 CONCLUSION

The mechanical strength of extruded WE43 was sufficient for mid‐facial application. Absorption of WE43 showed disadvantageous gas formation, but it was thought that the absorption rate could be optimized with a surface treatment such as coating. Plates and screws made with appropriately treated WE43 have the potential to be useful clinically.

## CONFLICT OF INTEREST

The authors declare no potential conflicts of interest with respect to the authorship and/or publication of this article.
